# Comparison of two European paediatric emergency departments: does primary care organisation influence emergency attendance?

**DOI:** 10.1186/s13052-017-0339-y

**Published:** 2017-03-08

**Authors:** F. Poropat, P. Heinz, E. Barbi, A. Ventura

**Affiliations:** 1 University of Trieste, IRCCS-Burlo Garofolo Trieste (Italy), via dell’Istria 65/1, Trieste, 34137 Italy; 2 Emergency Department, Addenbrooke’s Hospital, Cambridge University Hospitals NHS Foundation Trust, Cambridge, UK; 3 Institute for Maternal and Child Health - IRCCS “Burlo Garofolo”, Trieste, Italy

## Abstract

**Backround:**

To compare paediatric Emergency Department (ED) attendances and admission outcomes in two European hospitals with different paediatric primary care set-up.

**Methods:**

This is a retrospective prevalence study comparing all paediatric ED attendances during calendar years 2013 in two EDs with similar catchment area: one in Italy (Trieste) where paediatric primary care is provided by office paediatricians, the other, in the UK (Cambridge), where paediatric primary care is provided by general practitioners. Data on reason for presentation, discharge diagnosis and admission rate were collected and sub-group analysis for specific age groups (<1 year, 1–4 years, 5–15 years) was performed.

**Results:**

Over 12 months, 20.331 children (0–15 years old) were seen in Cambridge and 18.646 in Trieste, with a very similar age distribution in both centres, except for the youngest age group: the percentage of infants seen in comparison with the total number of children attending ED was 1/3 higher in England than in Italy (15.4% vs 11.4%). The reasons for attendance were similar: under 1 year of age, the chief complaints were fever, breathing difficulties and gastrointestinal problems while in the older age groups trauma represented the commonest reason. Among discharge diagnoses, no differences were found between the two hospitals, except for faltering growth and “well child”, more frequently diagnosed in English children under 5 years. The proportion of admissions was three times higher in Cambridge (14.1% vs 4.8%) with most children being admitted for infectious diseases.

**Conclusions:**

ED attendances in infants are more common in a primary care setting provided by general practicioner and, moreover, admission rates in all age groups are 1/3 reduced by primary care based paediatricians. Due to the methodological limits of this study, it isn't possible to evaluate whether these results depend only on paediatric primary care set-up or be determined by other confounding factors. New studies are needed to confirm this preliminary evidence.

## Background

In 1978, the Alma-Ata declaration, signed by 134 nations and 67 international organisations, defined primary care, particularly mother and child care*,* as the key tool for high quality and high equity healthcare services and proposed standards [1]. More than 30 years later, some goals of that declaration have still not been achieved [2]. There has been an increasing awareness and concern about the wide variation in healthcare for children and adolescents between countries at the primary care level. In a recent investigative study on the paediatric primary care (PPC) organisation in Europe [3], van Esso identified three different healthcare delivery systems: paediatrician based (in seven of 29 nations assessed), general practitioner based (in 12 out of 29) and a combined service with co-existence of paediatric primary care based services delivered by paediatricians and general practitioners (in 10 out of 29). It has been recognised that many factors, economic, geographical, and historical, contribute to these variations. No studies have shown which system is better in terms of efficacy and efficiency. There is an objective difficulty to identify appropriate outcomes to allow a comparison among different systems: conventional health indicators, such as neonatal and infant mortality or low birth-weight, are more related to income per capita than to primary care organisation, whilst more general indicators, such as national coverage of PPC, accessibility of services or user satisfaction, lack in specificity [4].

Recently, several authors have raised doubts about the capability of general practitioners (GPs) to continue acting as gatekeepers for paediatric patients because in some countries with general practitioner based system a higher demand on hospital emergency assessment and rate of inappropriate referral has been noted [5, 6].

The aim of this study was to compare paediatric Emergency Department (ED) attendances in two hospitals belonging to diverse PPC systems, the British general practitioner based system and the combined service of Italy.

## Methods

### Study design and setting

This was a retrospective prevalence study comparing all paediatric ED attendances during calendar years 2013 in two European hospitals with different PPC set-up. Both are university hospitals with comparable geographical catchment area and number of attendances in ED (approximately 20.000 children/year) and with comparable attraction rate from neighbouring hospitals, being both tertiary care specialist centres.

In both settings, medical care is free at the point of need, although in Italy children older than 14 years have to pay a fee if a health professional in ED considers the attendance as inappropriate. There are three ways to access ED: when parents take their child directly, by doctor’s referral or when brought in by emergency services in urgent cases.

#### Addenbrooke’s Hospital (Cambridge University Hospitals NHS Foundation Trust)

Addenbrooke’s Hospital is the largest centre providing secondary and tertiary paediatric services in the East of England. Paediatric ED functions as a dedicated area within a large adult ED and cares for children up to their 16th birthday with a catchment population estimated near to 52.000 (9,5% in the age band under 1 year old, 31.5% 1–4 years old, 30% 5–9 years old, 30% 10–15 years old) (http://cambridgeshireinsight.org.uk/populationanddemographics/districtreports), on the basis of a catchment area comprising Cambridge City, South and East Cambridgeshire Districts, though many children also attend from neighbouring counties.

Paediatric medical assessment of children is delivered jointly by paediatricians of all levels of seniority, adult emergency medicine physicians (EMP) and in the case of minor trauma patients also by Emergency Nurse Practitioners. Children can be diverted from the ED to an out-of-hours GP service based in the ED. Addenbrooke’s hospital does not have a separate paediatric admissions unit and the ED is the only entry point except for elective admissions and inter hospital transfers. Possible disposal outcomes are overnight in-patient admission, admission for short term observation during the times of 08.00 to 21.00 and discharge home with possible next day review in an urgent clinic slot by a senior paediatrician.

GPs are the providers of PPC. GP consultations generally take place in medical centres (practice or surgery) all day long, from 8 a.m. to 6.30 p.m. with some surgeries offering extended opening hours during weekdays and a GP run out-of-hours service over night and on weekends. Other community based health providers like Health visitors (‘well child’ nurses for the under fives) as well as specialist community paediatric services (provided to children with special needs by community paediatricians which work together with other specialist health workers, such as (educational) psychologists, physiotherapists and speech therapists) do not usually refer patients to ED. For newborns (up to 28 days old), community midwives provide assistance to parents following discharge after childbirth and can refer babies directly to hospitals in case of medical concerns. At the time of the study, National Health Service (NHS) direct, a telephone and web-based helpline provided a 24/7 nurse-led advice and health information service (now replaced by NHS 111) [7].

##### Istituto di Ricovero e Cura a Carattere Scientifico (IRCCS) Burlo Garofolo

The IRCCS Burlo Garofolo is the tertiary university children’s hospital of Trieste, capital city of Friuli Venezia Giulia in Italy. Similar to Addenbrooke’s it covers a geographically large area, the nearest centre offering equivalent services being in Lubiana, capital city of Slovenia, almost 90 km away, and in Padova 150 km far away.

Paediatric ED is attended by children up to 18 years old and covers a catchment population of approximately 48.000 (8% in the age band under 1 year old, 31% 1–4 years old, 32% 5–9 years old, 29% 10–15 years old), the catchment area including Trieste and neighbouring cities (http://demo.istat.it/pop2013/index.html). Medical assessment is provided by paediatricians of all levels of seniority and nurses.

Following ED attendance the possible outcomes are discharge home, discharge with referral to a community based paediatrician and in-patient admission (including short term observation up to 36 h).

PPC is provided in the majority of cases by community based paediatricians (CP) and rarely by family doctors (FD) who are comparable to GP. For the first 6 years of life PPC is exclusively provided by CP, from this age onwards a family can decide to have the child under the care of a FD or continue to see a CP until the age of 14 years. However due to the lack of CP in some regions, the proportion of care delivered by FD may vary. In Friuli Venezia Giulia almost 85% of children are cared for by CP.

Community based doctors (CP and FD) provide consultations 5 days per week for 3–6 h per day, being available for telephone advice each morning until 10 a.m., except Sundays and bank holidays. An organisation called Primary Care Outpatient Unit, set up jointly by CP and FP also provides medical assistance during office hours in some areas. The NHS provides out-of-hours phone coverage as well as urgent home care to all patients, using moonlighting non-pediatrician physicians [8].

### End points

We analysed data of all ED attendances during a 12 month period in both hospitals, comparing reasons for attendance, discharge diagnoses and patient disposal for 3 age groups (less than 1 year old, between 1 and 4 years old, and 5 to 15 years).

Patient related information was extracted from the electronic patient episode data from the two centres.

The following were compared:

- Total number of attendances and their outcomes (discharge or hospitalisation) in relation to catchment area and age bands above.
- Reason for attendance focussing mainly on 5 items: fever, breathing difficulties (tachypnoea, cough, shortness of breath), gastrointestinal symptoms (vomiting and diarrhoea), rashes and trauma. Furthermore, a few other more age specific conditions were assessed (febrile convulsion, drug ingestion, syncope).
- Discharge diagnoses, focussing on the most common for each age group (bronchiolitis, wheezing, pneumonia, gastroenteritis, pulled elbow, transient synovitis, diabetic ketoacidosis) and “well child”.
- Diagnoses that led to in-patient admission.

### Statistical analysis

The statistical analysis was performed using the website OpenEpi19 (http://www.openepi.com).

Categorical variables have been presented as numbers or percentages. χ- square testing was applied to assess the differences between outcomes of children seen at the IRCCS and those seen at Addenbrooke's Hospital, considering as statistically significant a p-value of <0.05. In the small sample sizes constituted by less than 50 units we have used the Fisher's exact test, considering as statistically significant a *p*-value of <0.05.

## Results

### Number and outcomes of attendances

Over 12 months, there were 20,331 attendances in Cambridge, 413 of those (2%) needed a few hours of clinical observation and 2.479 (12.1%) were admitted as in-patients.

In Trieste, 20.290 attendances were seen in ED. Of this figure patients aged 16 years and over were subtracted, leaving 18,646 urgent assessments of which 422 (2.3%) needed short term observation and 484 (2.6%) were hospitalised.

For the purpose of comparing the two centres, we considered short term observation as an inpatient admission.

Sub-dividing the cohorts into age groups, the percentage of infants seen in comparison with total children attended in ED is 1/3 higher in Cambridge than in Trieste (15.4% versus 11.5%, *p* <0.01), even though the age distribution of catchment population is similar. This variation is not found in older age groups (34.2% versus 35.4% in 1–4 year old group and 50.3% versus 53% in 5–15 year old).

### Reason for attendance

In the youngest age group, the main symptoms reported by parents on admission to both hospitals were fever, breathing difficulties and gastrointestinal symptoms. The number of infants under 12 months attending with these problems in Cambridge is significantly higher (Table 1).

**Table 1:** Reason for attendance under 1 years old infants

	Cambridge	Trieste	*p*
Fever	*900 (28.6%)*	563 (26.1%)	0.05
Breathing difficulties	*644 (20.4%)*	345 (16%)	<0.01
Gastrointestinal symptoms	*390 (12.4%)*	214 (9.9%)	<0.005
Febrile rash	*193 (6.1%)*	79 (3.6%)	<0.001
Febrile seizure	15 (0.47%)	6 (0.27%)	ns
Trauma	251 (7.9%)	202 (9.3%)	ns
Total attendance under 1 year old	*3144*	*2149*	

In brackets: percentage of specific symptom out of total attendance. *ns* means not statistically significative

No such difference was found for attendances related to trauma and febrile seizures in this age group.

For children aged 1 to 4 years, the commonest reason for seeking medical assistance was trauma, together with fever and breathing difficulties (Table 2). Again the number in relation to local population was significantly higher in Cambridge.

**Table 2:** Reason for attendance in 1–4 year old children

	Cambridge	Trieste	*p*
Fever	*1916 (27.5%)*	1631 (24.6%)	<0.0001
Breathing difficulties	*814 (11.7%)*	428 (6.4%)	<0.0001
Gastrointestinal symptoms	580 (8.3%)	610 (9.2%)	ns
Febrile seizure	63 (0.9%)	53 (0.8%)	ns
Trauma	*2010 (28.8%)*	1350 (20.4%)	<0.0001
Ingestion/overdose	*203 (2.9%)*	142 (2.1%)	<0.01
Total attendance under 1–4 years old	*6957*	*6613*	

In brackets: percentage of specific symptom out of total attendance. *ns* means not statistically significative

In the oldest age group, trauma represented the most frequent reason for seeking medical assessment in both centres, albeit more commonly in Cambridge. There was no difference in the proportion of children presenting with infection related and respiratory problems presenting to both centres (Table 3).

**Table 3:** Reason for attendance in 5–15 year old children

	Cambridge	Trieste	*p*
Fever	796 (7.7%)	845 (8.5%)	ns
Breathing difficulties	355 (3.4%)	339 (3.4%)	ns
Gastrointestinal symptoms	390 (3.8%)	415 (4.1%)	ns
Trauma	*4381 (42.8%)*	4161 (42%)	<0.0001
Convulsion	*88 (0.8%)*	23 (0.2%)	<0.0001
Ingestion/overdose	*117 (1.1%)*	69 (0.6%)	<0.01
Faint	43 (0.4%)	56 (0.5%)	ns
Total attendance under 5–15 years old	*10231*	*9885*	

In brackets: percentage of specific symptom out of total attendance. *ns* means not statistically significative

In the age groups of 1 to 4 and 5 to 15 year olds, accidental ingestions and deliberate overdoses were more frequent in Cambridge. When focusing on the older end of the age spectrum, 79 of 117 presented in Cambridge were older than 13 years old compared to 26 out of 69 who were seen in Trieste. Among those, 32 English children (and only 2 Italians) had taken an overdose with suicidal intent.

### Diagnosis on discharge from ED

Gastroenteritis and bronchiolitis were the commonest diseases in infants seen at Addenbrooke’s and IRCCS respectively (Table 4). The diagnosis of faltering growth was more commonly made in Cambridge, as was the diagnosis of “well infant”, which was made 2,7 times more frequently than in Trieste.

**Table 4:** Discharge diagnoses in infants

	Cambridge	Trieste	*p*
Bronchiolitis	*318 (10.1%)*	178 (8.2%)	0.02
Wheezing	43 (1.3%)	45 (2%)	ns
Pneumonia	10 (0.3%)	3 (0.1%)	ns
Gastroenteritis	*329 (10.4%)*	115 (5.3%)	<0.001
Constipation	43 (1.3%)	29 (1.3%)	ns
Faltering growth	*80 (2.5%)*	17 (0.8%)	<0.001
Well infant	*281 (8.9%)*	72 (3.3%)	<0.001
Total attendance under 1 year old	*3144*	*2149*	

In brackets: percentage of specific diagnosis out of total attendance. *ns* means not statistically significative

No other differences among the remaining diseases considered were found.

In the age group of 1 to 4 year old, the only diagnosis significantly more frequently made in Cambridge was again that of a “well child” (Table 5).

**Table 5:** Discharge diagnoses in 1–4 year old children

	Cambridge	Trieste	*p*
Wheezing	251 (3.6%)	206 (3.1%)	ns
Pneumonia	87 (1.2%)	104 (1.5%)	ns
Gastroenteritis	580 (8.3%)	533 (8%)	ns
Ingestion/overdose	*203 (2.9%)*	142 (2.1%)	<0.001
Transient synovitis	42 (0.6%)	39 (0.5%)	ns
Pulled elbow	123 (1.7%)	134 (2%)	ns
Well child	*98 (1.4%)*	24 (0.3%)	<0.001
Total attendance in 1–4 years old	*6957*	*6613*	

In brackets: percentage of specific diagnosis out of total attendance. *ns* means not statistically significative

Focusing on common causes of ED attendance arguably not depending on PPC organisation, such as transient synovitis or pulled elbow, no variation was observed.

In the oldest age group, pneumonia was more commonly diagnosed at IRCCS, of those, nine pneumonias were complicated by empyema in Trieste and three in Cambridge (Table 6). Deliberate self harm was recorded 15.46 times more frequently as a diagnosis in Cambridge than in Trieste.

**Table 6:** Discharge diagnosis in 5–15 year old children

	Cambridge	Trieste	*p*
Wheezing	110 (1%)	130 (1.3%)	ns
Pneumonia	43 (0.4%)	*81 (0.8%)*	<0.007
Gastroenteritis	390 (3.8%)	415 (4.1%)	ns
Transient synovitis	19 (0.1%)	15 (0.1%)	ns
Diabetes onset	21 (0.2%)	18 (0.1%)	ns
*Diabetic ketoacidosis*	11 (0.1%)	8 (0.08%)	ns
Self harm	*32 (0.3%)*	2 (0.02%)	<0.,001
Total attendance in 5–15 years old	*10231*	*9885*	

In brackets: percentage of specific diagnosis out of total attendance. *ns* means not statistically significative

### Reasons for inpatient admission

Admissions as a the ratio of attendances per diagnosis and hospitalisations differed significantly between the two hospitals. Overall the proportion of children being admitted was three times higher for Addenbrooke’s compared to IRCCS (14.1% vs 4.8%).

In the youngest group, the conversion rate was 24.1% of all attendances in Cambridge compared to 9.4% in Trieste. Bronchiolitis was the most common reason for admission in both hospitals, but as a proportion, children with this diagnosis were much more likely to be admitted in Cambridge: 318 infants had a diagnosis of bronchiolitis and 138 of those were hospitalised in Addenbrooke’s (43.3%) compared to 30 patients out of 178 (16.8%) at IRCCS. A similar imbalance was found for pneumonia and gastroenteritis. No case of jaundice was admitted in Trieste, in contrast to almost half of the infants attending with this diagnosis in Cambridge resulting in hospitalisation. However, at IRCCS, many more patients with cranial trauma and febrile seizures were hospitalised (Table 7).

**Table 7:** Reason for admission in the under 1 year old group

	Cambridge (%)	Trieste (%)	*p*
Bronchiolitis	*138 (43.3%)*	30 (16.8%)	<0.001
Wheezing	5 (11.6%)	4 (8.8%)	ns
Pneumonia	*8 (80%)*	1 (33.3%)	<0.0001
Gastroenteritis	*126 (38.2%)*	15 (13%)	<0.0001
Cranial trauma	9 (4.3%)	*14 (10.5%)*	0.02
Febrile siezure	2 (13.3%)	*5 (83.3%)*	<0.0001
Jaundice	*103 (51.5%)*	0 (0%)	<0.0001
Total admissions under 1 year old	*758*	*203*	

In brackets: percentage of specific diagnosis out of total admissions. *ns* means not statistically significative

Also in the older age groups, the percentage of admission for infection related diagnoses was significantly higher in Addenbrooke’s (Table 8).

**Table 8:** Reasons for admission in 1–4 year old and 5–15 year old

	Cambridge (%)	Trieste (%)	Cambridge (%)	Trieste (%)
Wheezing	*68 (27%) ϑ*	10 (4.8%)	33 (30%) ϑ	9 (6.9%)
Pneumonia	*30 (34.4%) ϑ*	8 (7.6%)	*32 (66.6%) ϑ*	21 (25.9%)
Gastroenteritis	*80 (13.7%) ϑ*	20 (3.7%)	*38 (9.7%)*	28 (6.7%)
Cranial trauma	37 (8.2%)	28 (8.2%)	22 (4.5%)	*57 (19.7%) ϑ*
Ingestion/overdose	55 (27%)	28 (19.7%)	*70 (59.8%) ϑ*	10 (14.4%)
Febrile seizure	14 (22.2%)	*26 (49%) ϑ*		
Total admissions for age groups	873	*247*	*1180*	*456*

In brackets: percentage of specific diagnosis out of total admissions for each age groups. ϑ: *p* <0.01

Ingestions and overdoses in the oldest age group were also admitted more frequently in Addenbrooke’s, the proportion of deliberate self harm already having been mentioned. This contrasts with a pattern of higher hospitalisation rates due to head injury and febrile seizure in Trieste.

## Discussion

In this retrospective study we wanted to compare the paediatric ED work load in two hospitals with different PPC models to evaluate the gate-keeper function of different primary care set-up.

Whilst an increase in paediatric ED attendances is seen across most of Europe, we were surprised by the wide variation of attendances of infants in the two emergency departments representative of English and Italian situation. Being comparable in relation to catchment area and services provided, Addenbrooke’s hospital sees per annum almost 1/3 more infants than Burlo’s hospital. Intriguingly, the diagnosis of “well child” was 2,7 times more frequent in Cambridge than in Trieste. Ambulatory care sensitive conditions, such as failure to thrive, gastroenteritis and viral rash, conditions that often develop gradually and could be managed in an appointment based encounter were much more commonly seen in Cambridge. Conversely, no differences were found for those problems of a sudden onset or rapid worsening of clinical condition at this age, such as pneumonia or wheezing. We believe that a paediatrician’s presence in the community setting could explain the dissimilarity, because apart from having experience in assessing medical problems of high acuity, the management skills and experience in relation to the youngest age group exceed that of a FD. Previous studies have revealed a link between rate of admissions and PPC organisation, especially in the first years of life, independently of other confounding variables such as economic condition of the family, parental level of education, social environment, accessibility of community doctor [9–12].

After the first year of life, the difference in attendances in each age group tends to disappear progressively and, except for certain diagnoses, only a few differences remain. In effect this could be easily explained by the fact that the two PPC-systems don’t differ so much when it comes to the care of older children: in Italy FD can provide care for children older than six and a recent national study has shown that only 40% of the population up to 14 years old attended a CP.

Moreover, it is worthwhile to point out that ambulatory care sensitive conditions form the majority of all attendances in both hospitals. This may represent an inappropriate use of hospital services in most cases and, indirectly, a failure of both PPC services, as already shown in previous studies [13, 14].

Injuries were seen more frequently in Addenbrooke’s ED than in Burlo’s hospital, possibly due to the fact that Addenbrooke’s is the regional trauma centre and attracts trauma patients from across the region.

Similarly, psychiatric and neurological problems, such as epileptic seizures and self harm, were supposedly seen less frequently in Trieste because there are two other hospitals in Friuli Venezia Giulia who take care of children with neuropsychiatric problems. It is therefore plausible that these hospitals attract a proportion of these patients.

Finally, the hospitalisation rate, the proportion of children whose attendance results in an in-patient admission was more than three times higher in Addenbrooke’s than IRCCS Burlo Garofolo. This was a surprise finding as the guidelines for the commonest diseases (bronchiolitis, wheezing, pneumonia, gastroenteritis) and admission criteria are quite similar. Despite that, considering the whole cohort, hospitalisations due to bronchiolitis and gastroenteritis happened more often in Cambridge (43.3% vs 16.8% and 13.1% vs 5%) and for pneumonia were almost three times as common (40% vs 15%). It is unlikely that diseases seen in the Cambridge ED were more severe and the patients needed more aggressive treatment. It would have been useful to compare the length of stay but the study design did not allow us to review this data. Several publications have shown a rise in emergency hospital admissions in England for conditions usually managed in the community [15, 16]. The emergency admission rate, based on analysis of Hospital Episode Statistics and population estimates for England between 1999 and 2010, has increased by 28%. Considering only infants it found a rise of 52%. Admission rates for upper respiratory tract infections rose by 22%, lower respiratory tract infections by 40% and gastroenteritis by 31%. The authors attributed this trend to several conditions: the recent organisational changes in the NHS, new models of acute paediatric care, such as short-stay units, and a decrease in willingness of parents and carers of children to tolerate uncertainty [17, 18].

It may also be speculated that the availability of a CP rather than an adult practicioner is a factor of reassurance for the ED paediatrician and the families and hence facilitates immediate discharge and follow up of children, also in spite of the wide difference of office hours availability between nations, only 3–6 h per day in Italy versus almost 10 h in England.

Furthermore whilst IRCCS Burlo Garofolo is staffed by paediatricians only, in Cambridge adult physicians also care for children, although the decision to admit is usually made by a paediatrician.

Only two reasons for admission in our study have been more frequent at Burlo’s hospital: febrile convulsions and head injury. For the first condition, the two hospitals have different guidelines. Whilst in Trieste patients are admitted for a short observation, children in Cambridge are promptly discharged. The same cannot be said for the management of head injury, because the guidelines are identical. To better understand this dissimilarity, it would be interesting to compare imaging performed in both hospitals to see if the prudent approach of Italian ED doctors with a much higher hospitalisation rate is linked to a comparatively lower rate of CT scans performed.

We are aware that there are several limitations in this study. First of all, we have been able only to estimate the catchment population of two hospitals, considering data from hospital statistical registry. Meanwhile no statistical differences have been found among admissions due to urgent diseases typically seen in ED, such as febrile convulsion, pulled elbow or diabetic ketoacidosis, and for this reason, indirectly, it could be speculated that catchment area is comparable. Secondly, the data was only collected in two centres which may not be representative of the relevant national practice. Likewise, in Italy, the hospitalisation rates range from 63/1000 children resident in Friuli Venezia Giulia to 101/1000 children resident in the rest of Italy. Other variables which could influence health seeking behaviours and outcomes, such as parent factors, access to primary care, economic and social background, re-attendance rate as well as seniority and training of medical staff at various times of the day, were all not assessed. Finally, as we did not undertake an economic analysis of both PPCs, a true assessment of effciency and cost effectiveness cannot be made.

## Conclusion

This study has shown a significantly higher attendance rate of infants in a hospital with primary care delivered by a FD based PCC in comparison to a system where access to a paediatrician is the rule. It has been also found a rate of hospitalisation for common diseases in all age groups three times higher. However the study design hasn’t allowed us to rule out if there were other factors which could have influenced our results and hence new studies are needed to clarify our findings. Not being able to provide a definitive answer, this paper contributes at the very least to the still growing debate on what should be the role of primary care paediatricians in Italy and abroad.

## Abbreviations

PPC: Paediatric primary care
GP: General practitioner
NHS: National Health Service
ED: Emergency Department
EMP: Emergency medicine physicians
FD: Family doctor
CP: Community based pediatricians
